# Transcriptomic Analysis of Dibenzofuran Degradation by *Burkholderia* sp. FM-2 Under Cd(II) Stress

**DOI:** 10.3390/microorganisms14061297

**Published:** 2026-06-09

**Authors:** Xiuwei Hou, Lei Huang, Xintong Duan, Ying Zhai, Xin Zhao, Meitong Li

**Affiliations:** 1 Tianjin Key Laboratory of Organic Solar Cells and Photochemical Conversion, College of Chemistry and Chemical Engineering, Tianjin University of Technology, Tianjin 300384, China; 20240957@stud.tjut.edu.cn (X.H.); huanglei@tjut.edu.cn (L.H.); duanxintong@stud.tjut.edu.cn (X.D.); xiaozhai@stud.tjut.edu.cn (Y.Z.); 2 School of Resources and Civil Engineering, Northeastern University, Shenyang 110004, China

**Keywords:** *Burkholderia* sp., biodegradation, dibenzofuran, heavy metal, transcriptome analysis

## Abstract

Co-contamination with dibenzofuran (DBF) and cadmium (Cd(II)) poses a major challenge in environmental remediation. While *Burkholderia* sp. can degrade polycyclic aromatic hydrocarbons and tolerate heavy metals, the coordinated mechanism governing DBF degradation under high Cd(II) stress remains elusive. Here, we characterize *Burkholderia* sp. FM-2, which optimally degrades 600 mg/L DBF at pH 6.0 and 25 °C, achieving 91.8% removal within 48 h. FM-2 exhibits exceptional Cd(II) tolerance, with a minimum inhibitory concentration of 2000 mg/L. UPLC-MS/MS confirms DBF degradation via dioxygenase-mediated hydroxylation and sequential enzymatic reactions. Transcriptomics reveals, for the first time, concurrent upregulation of genes encoding RND efflux pumps, ABC transporters, P-type ATPases, and core DBF-degrading enzymes under high Cd(II) stress, enabling the synergistic maintenance of intracellular Cd(II) homeostasis and efficient DBF degradation. Collectively, FM-2 remediates DBF-Cd(II) co-contamination via coordinated transcriptional regulation of degradation and detoxification pathways, offering a promising strain resource and molecular basis for the bioremediation of co-contaminated environments.

## 1. Introduction

Polycyclic aromatic hydrocarbons (PAHs) are persistent organic pollutants containing two or more benzene rings [1]. They are widely distributed in various environmental media [2,3] and pose severe threats to ecological security and human health due to their high toxicity, lipophilicity, and carcinogenicity [4]. As a model compound for studying the degradation of polycyclic aromatic hydrocarbons and dioxins, dibenzofuran (DBF) is primarily derived from anthropogenic incomplete combustion, and industrial by-products, with significant variations in DBF contamination levels across these sources: DBF concentrations in pulp bleaching effluent are in the ng/L range [5], whereas coal tar processing and creosote sites may result in groundwater concentrations as high as 150 μg/L [6]. Incineration and thermal treatment processes primarily release DBF via flue gas emissions, with emission factors ranging from 22 to over 1000 ng I-TEQ/t, depending on the specific process [7]. DBF is frequently detected in contaminated water and soil, where it can bioaccumulate through the food chain and cause persistent environmental and health risks. Moreover, in actual contaminated environments, persistent organic pollutants such as DBF with strong bioaccumulation and teratogenic toxicity often form co-contaminated systems with heavy metals (HMs) including lead, cadmium, mercury, and zinc, significantly increasing the difficulty of remediation.

Heavy metals (HMs) are a category of inorganic pollutants. Unlike organic compounds, they cannot be degraded naturally. In environmental systems, heavy metals primarily adapt to environmental changes by transforming their existing chemical speciation. Typical examples include the conversion of Hg^2+^ to less toxic Hg^0^ [8], As^3+^ to As^5+^ [9], and Cr^6+^ to Cr^3+^ [10]. Although trace amounts of ions such as zinc and iron are essential for human physiological activities, elevated concentrations can trigger severe toxic effects, including reproductive malformations, renal dysfunction, respiratory distress, cellular mutation, and carcinogenesis [11]. For instance, cadmium binds to proteins and causes cellular damage, while iron exerts corrosive effects and participates in lipid peroxidation [12]. In addition, the interaction between heavy metals, proteins, and deoxyribonucleic acid (DNA) induces the production of reactive oxygen species (ROS) and further triggers oxidative stress. Such stress leads to base damage and DNA strand breakage, alters cell morphology, and accelerates cell apoptosis [13]. The combination of HMs and polycyclic aromatic hydrocarbons (PAHs) promotes the formation of heavy metal–organic pollutant complexes through cation–π interactions. Meanwhile, heavy metals exert irreversible inhibition on microbial metabolic enzymes, thereby suppressing microbial growth and metabolism and ultimately weakening the microbial degradation capacity toward DBF [14]. Therefore, research on synergistic remediation technologies for PAH-HM co-contamination is of great theoretical and practical significance to advance the field of contaminated environmental remediation.

Numerous studies have focused on the toxic mechanisms and remediation technologies of single polycyclic aromatic hydrocarbons (PAHs) or heavy metals (HMs). Advanced oxidation, adsorption, electrochemical remediation, composting and other techniques have been successfully applied to the treatment of single pollutants [15]. However, due to the essential differences in physicochemical properties between PAHs and HMs, most remediation methods suitable for single contamination exhibit insufficient performance in combined pollution systems. For instance, electrokinetic remediation, which is efficient for heavy metal removal, and conventional physical and chemical methods for dibenzofuran (DBF) elimination, tend to produce toxic by-products and pose high risks of secondary pollution. In this context, microbial biodegradation is regarded as an eco-friendly and sustainable approach to remediate environmental media co-contaminated with DBF and heavy metals. A variety of dominant microbial strains for combined remediation have been screened at present, most of which are bacteria and a small proportion are fungi. Representative strains include *Pseudomonas fluorescens* [16], *Sphingomonas* sp. [17], and *Rhodococcus* sp. [18], and several of these strains exhibit favorable degradation potential. At present, the interaction mechanisms between HMs and DBF remain poorly understood. Existing evidence indicates that heavy metals exert inhibitory effects on the microbial degradation of DBF. Heavy metal ions can markedly weaken the DBF degradation capacity of functional strains by inhibiting the activity of microbial metabolic enzymes [14], damaging cell membrane integrity [19], and inducing excessive accumulation of reactive oxygen species [20]. Accordingly, it is necessary to further explore and optimize the synergistic remediation strategies for combined PAHs and heavy metal pollution.

Transcriptomics technology enables high-throughput analysis of gene expression profiles at the whole-genome level. Through metatranscriptomic analysis, it is possible to identify which genes are activated under specific conditions and to elucidate the functional roles of specific microorganisms. For example, Li et al. [21] identified differentially expressed genes of the ABC transporter family through transcriptomic analysis in a study on the response of *Burkholderia* sp. JT-M8 to the combined stress of cadmium and PAHs. They confirmed that the transcriptional activation of these genes is critical for the strain to maintain degradation efficiency and alleviate heavy metal toxicity. In the research on the bioaccumulation of *Burkholderia* cepacia GYP1 under cadmium stress conducted by Zhang et al. [22], in-depth transcriptomic analysis revealed that Cd(II) could upregulate the expression of cadmium/zinc efflux ATPase, type VI protein secretion system, and glutathione-S-transferase related to cadmium response. These regulatory mechanisms contribute to the maintenance of intracellular cadmium homeostasis. Despite these insights, the specific transcriptional responses of *Burkholderia* strains to combined DBF and Cd(II) stress, as well as differences in the expression of transporter-encoding genes, remain largely unexplored.

This study utilized the *Burkholderia* FM-2 strain, which had been previously screened and isolated by the laboratory and possesses a high capacity for the degradation of PAHs. Previous genomic and transcriptomic analyses confirmed that FM-2 possesses a specialized PAH degradation gene cluster and exhibits significant degradation capacity for naphthalene, fluorene, dibenzofuran and dibenzothiophene, with degradation efficiency for these substrates exceeding 58% [23]. However, almost all existing reports on FM-2 have focused on its performance under single PAH contamination [23,24]; no study has systematically evaluated its DBF degradation behavior under heavy metal stress, nor has any research been conducted at the transcriptomic level to investigate the underlying molecular regulatory mechanisms. To address this gap, this study optimized the degradation conditions for strain FM-2 using DBF as the sole carbon source and employed transcriptomic analysis to investigate the patterns of differential gene expression under Cd(II) stress. This study overcomes the limitations of single-pollutant remediation and demonstrates for the first time that *Burkholderia* FM-2 can simultaneously and efficiently address DBF and Cd(II) co-contamination. It elucidates the molecular regulatory mechanisms of microorganisms under combined stress, thereby revealing their potential for application in DBF-Cd(II) co-contaminated systems. Second, transcriptomic approaches identified key functional genes responsive to Cd(II) stress and elucidated the cooperative regulatory network of multiple genes and pathways involved in DBF degradation under Cd(II) stress. This provides a new method and theoretical basis for addressing combined DBF and heavy metal pollution.

## 2. Materials and Methods

### 2.1. Chemical Reagents and Media

DBF (purity 98%) and other reagents were of analytical grade, sourced from Tiangen Biochemical Co., Ltd. (Beijing, China).

Inorganic salt medium (g/L): (NH_4_)_2_SO_4_ 3.96, Na_2_HPO_4_ 1.50, KH_2_PO_4_ 3.48, MgSO_4_ 0.70, yeast powder 0.01; pH 7.0–7.2; add 2% glycerol into the inorganic salt medium at the time of inoculation to form the seed liquid medium.

LMM medium (g/L): KH_2_PO_4_ 0.1, Na_2_HPO_4_ 0.1, NH_4_NO_3_ 0.5, (NH_4_)_2_SO_4_ 0.5, MgSO_4_ 0.2, CaC1_2_ 0.02, FeCl_2_ 0.002, and MnSO_4_ 0.002, deionized water 1 L, prepared in deionized water and pH adjusted to 6.5.

LB medium (g/L): yeast powder 5.0, peptone 10.0, NaCl 10.0, pH 7.0–7.2. For solid medium, agar was supplemented at 20 g/L.

Degradation medium: DBF was dissolved with hexane to form a mother liquor, filtered and sterilized by 0.22 μm organic filter membrane, and then added into the sterilized inorganic salt medium according to the appropriate concentration to form the degradation medium.

### 2.2. DBF Degradation Experiment

#### 2.2.1. Optimization of DBF Degradation Conditions

Following a screening procedure in our laboratory, the bacterial strain *Burkholderia* sp. FM-2 was obtained from hydrocarbon-contaminated soil at a Xinjiang oilfield site in China [25]. FM-2 was cultured to logarithmic stage in seed liquid medium, transferred to DBF degradation medium at 2% inoculum, and then we analyzed the growth and DBF degradation of FM-2 under different DBF addition (300–800 mg/L), degradation cycles (0–72 h), temperatures (15–35 °C), pH (5–10), and NaCl concentration (0–2.5%). The proliferation of the microbial and decomposition of DBF under the environmental conditions were measured.

Hexane containing the internal standard hexamethylbenzene was used to extract the DBF degradation culture medium three times in a 1:1 ratio, and the three organic phases were combined. The mixture was dried over anhydrous Na_2_SO_4_ and the solvent was removed by vacuum distillation. The residue was dissolved in n-hexane and analyzed by gas chromatography (GC) to determine the percentage of DBF degradation. Three parallel experiments were conducted for each group, with a blank control. Column: Agilent HP-5 (30 m × 320 μm × 0.25 μm), detector: FID detector. Parameter settings: temperature 300 °C; column temperature: 80 °C maintained for 3 min, and then increased to 290 °C at 5 °C/min; helium was used as the carrier gas; inlet temperature of 250 °C; interface temperature of 280 °C.

The bacteria were centrifuged and collected, and the biomass of FM-2 was determined by measuring the absorbance value at 600 nm using a UV–visible spectrophotometer.

The DBF degradation efficiency was calculated according to the following formula:

DBF degradation efficiency (%) = (C_0_ − C_t_)/C_0_ × 100%

where C_0_ represents the initial concentration of DBF in the reaction system, and C_t_ is the residual concentration of DBF after cultivation for time t.

#### 2.2.2. UPLC-MS/MS Analysis of Metabolites

Media of DBF degradation after 12, 24, and 36 h were taken. Samples were taken out three times and the volume of the supernatant was taken. The dry powder was dried using anhydrous sodium sulfate and then the powder was left and nitrogen-blown to almost dryness. Sample preparation: The dried extract was redissolved to HPLC-grade acetonitlifted and filtered (0.22 μm). Chromatographic conditions: Isocratic elution using a mobile phase consisting of methanol:water (70:30, *v*/*v*) at a flow rate of 0.5 mL/min. Detection UV at 254 nm combined with mass spectrometric analysis.

### 2.3. Effect of Heavy Metal Cd(II) in LMM on DBF Degradation

CdCl_2_ solution (sterilized by filtration through a 0.22 μm membrane filter) was used to determine the minimum inhibitory concentration (MIC) of Cd(II) to evaluate the heavy metal resistance of degrading strain FM-2. For the experimental group, different concentrations of Cd(II) (0–2000 mg/L) were added into 30 mL LMM medium supplemented with 600 mg/L DBF. Subsequently, 2% (*v*/*v*) seed culture of strain FM-2 in the exponential growth phase was inoculated, followed by continuous shaking incubation at a constant temperature of 25 °C for 7 days in a shaker. Three control groups were established in parallel: Control I contained strain FM-2 and heavy metals without DBF interference; Control II contained strain FM-2 and DBF without heavy metal addition; Control III served as a sterile blank control containing both heavy metals and DBF but no strain FM-2.

#### 2.3.1. Scanning Electron Microscopy Energy-Dispersive X-Ray Spectroscopy (SEM/EDS) Analysis

A Cd(II) concentration of 100 mg/L was selected for subsequent experiments. Strain FM-2 was cultured in LMM medium supplemented with 100 mg/L Cd(II) and 600 mg/L DBF at 25 °C with constant shaking for 7 days. LMM medium without metal ions was set as the control. After incubation, the bacterial suspension was centrifuged, and the collected cells were rinsed three times with deionized water. The washed biomass was fixed in 2.5% glutaraldehyde for 30 min, followed by 2–3 rinses with deionized water and sequential ethanol gradient dehydration. Finally, the samples were vacuum freeze-dried, and SEM-EDS was applied to characterize the surface morphological changes of strain cells after heavy metal adsorption.

#### 2.3.2. Fourier Transform Infrared Spectroscopy (FTIR) Analysis

Cultivation was performed with shaking at 25 °C and 200 rpm for 1, 3, 5, and 7 days, respectively, in the above manner to evaluate heavy metal bioaccumulation. The cultured bacterial solution was centrifuged at 8000 r/min for 10 min. The obtained bacterial pellets were rinsed three times with deionized water and freeze-dried at low temperature using a vacuum freeze dryer. The heavy metal-containing samples were mixed with dried KBr at a mass ratio of 1:100, and spectral measurement was conducted with a Perkin Elmer FTIR spectrometer.

#### 2.3.3. Inductively Coupled Plasma-Emission Spectrometer (ICP-OES) Analysis

Strains were cultured in the above manner. Strain FM-2 was inoculated into LMM medium containing 200 mg·L^−1^ Cd(II) and 600 mg·L^−1^ DBF to evaluate heavy metal bioaccumulation. Subsequent experimental operations were carried out referring to the method described by Liu et al. [25]. The extracellular supernatant (S1) and intracellular supernatant (S2) were separated in different 50 mL centrifuge tubes. An inductively coupled plasma mass spectrometer was used to analyze S1 and S2 to determine the heavy metal contents in the extracellular (S1) and intracellular fractions (S2).

### 2.4. Transcriptomic Analysis of Burkholderia *sp.* FM-2 Under Cd(II) Exposure

Bacterial cultures in the logarithmic growth phase were inoculated at a volume ratio of 2% into LMM medium containing 600 mg/L DBF, supplemented with a Cd(II) concentration of 100 mg/L. The cultures were incubated at 25 °C and 200 rpm for 7 days. After incubation, the bacterial solution was centrifuged at 10,000 rpm for 10 min at 4 °C to collect cell pellets. The precipitated cells were rinsed three times with pre-cooled PBS buffer, and the supernatant was discarded. The harvested cells were frozen in liquid nitrogen for 10 min and then stored at −80 °C for subsequent RNA extraction. The inorganic salt medium containing only strain FM-2 and DBF without heavy metal addition was set as the blank control. Three biological replicates were established for both the experimental group and the control group.

Transcriptome sequencing was performed by Majorbio (Shanghai, China) using the NovaSeq X Plus platform (Illumina, Inc., San Diego, CA, USA). The company employed a standard prokaryotic transcriptome sequencing workflow. Firstly, total RNA was extracted using the RNAprep Pure Cell/Bacteria Kit (Tiangen, Beijing, China) in accordance with the manufacturer’s instructions. The concentration and purity of the extracted RNA were determined using a Nanodrop 2000 (Thermo Fisher Scientific, Waltham, MA, USA); RNA integrity was verified by agarose gel electrophoresis, and the RNA integrity number (RIN) was measured with an Agilent 2100 Bioanalyzer (Agilent Technologies, Santa Clara, CA, USA). Subsequently, ribosomal RNA (rRNA) was depleted from total RNA using the Ribo-Zero™ Plus Kit (Thermo Fisher Scientific, Waltham, MA, USA) to enrich mRNA. The enriched RNA was then fragmented reverse-transcribed (using random primers) and amplified by bridge PCR to construct an Illumina sequencing library. Finally, the constructed library was sequenced using the PE150 protocol on the Illumina NovaSeq X Plus platform (Illumina Inc., San Diego, CA, USA). All other bioinformatics analyses were performed in accordance with the methods described by Ma et al. [26].

### 2.5. Quantitative Real-Time PCR (qRT-PCR) Analysis

To further validate the differentially expressed genes, we selected five candidate transporter genes identified in the FM-2 strain via transcriptomic sequencing. The primers used for this validation are documented in Table S1. qRT-PCR assays were run on a Q9600 series real-time fluorescence quantitative PCR instrument DEG (Bio-Gener Technology Co., Hangzhou, China). The 2× RealStar Fast dye-based qPCR premix (GenStar, Beijing, China) was used as follows: 10 μL of 2× RealStar Fast SYBR qPCR-premix, 0.5 μL of forward primer (10 μM), 0.5 μL of reverse primer (10 μM), 0.4 μL of reverse primer (10 μM), 0.5 μL of reverse primer (10 μM), 0.4 μL High/Low ROX Reference Dye, and 1 μL DNA template. The optimal cycling conditions were pre-denaturation at 95 °C for 2 min, followed by 40 cycles of amplification, with each cycle including denaturation at 95 °C for 15 s, annealing at 54 °C for 20 s, and extension at 72 °C for 30 s; fluorescence signals were collected at the extension stage throughout the whole process. The 2^−ΔΔCt^ method was used to calculate the relative expression levels of the target genes. All samples were run independently three times.

### 2.6. Statistical Analysis

Statistical significance of the inter-group comparisons was measured by means of Student *t*-tests and one-way ANOVA, followed by the post hoc analysis with the Tukey–Kramer test. The criterion of statistical significance set was set to a *p*-value less than 0.05. All quantitative values are described as means + standard deviation (SD). In order to guarantee methodological strength and reproducibility, three independent replicates were applied to every experiment.

## 3. Results and Discussion

### 3.1. Dibenzofuran Degradation by Burkholderia *sp.* FM-2

#### 3.1.1. Effect of Culture Conditions on DBF Degradation of Strain FM-2

**Figure 1:**
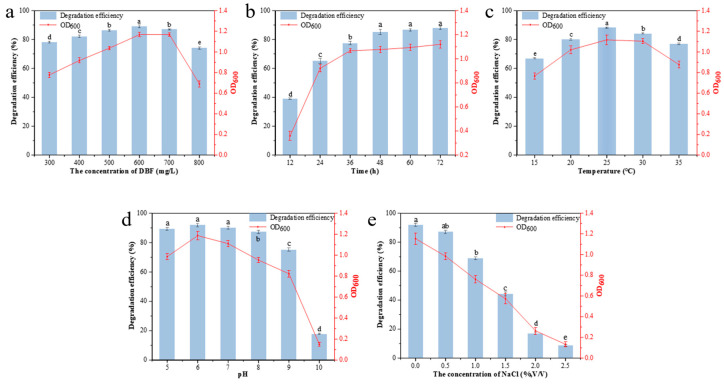
Effects of environmental conditions on DBF degradation and cell growth. (**a**) Concentration of DBF; (**b**) time; (**c**) temperature; (**d**) pH; (**e**) concentration of NaCl. The data presented in the graph are the mean ± SD values of replicates. The different letters above the bars indicate significant differences among the different groups (*p* < 0.05, *n* = 3). Statistical significance was determined using Tukey–Kramer test.

This experiment aimed to explore the effects of multiple factors (initial DBF concentration, incubation time, temperature, pH and salinity) on DBF degradation. As shown in Figure 1a, the results indicate that strain FM-2 could efficiently degrade DBF at concentrations ranging from 300 to 800 mg/L, with a degradation efficiency all exceeding 70%. When the initial DBF concentration was 600 mg/L, the maximum degradation efficiency of 89% was achieved under the conditions of 25 °C, 200 rpm, and 72 h of shaking incubation (pH 7.2, without NaCl addition). The degradation efficiency began to decrease once the DBF concentration exceeded 600 mg/L; therefore, 600 mg/L DBF was selected as the baseline concentration for subsequent culture condition optimization. Further experiments demonstrated that in the inorganic medium with 600 mg/L DBF as the sole carbon source, the DBF degradation efficiency was positively correlated with bacterial growth with prolonged incubation time. During the period of 0–48 h, the strain remained in the logarithmic growth phase with a rapid increase in degradation efficiency. The degradation efficiency reached 85.1% at 48 h, with a degradation efficiency of 0.024 mmol/(L·h), which was significantly higher than that of most previously reported DBF-degrading strains. For example, the DBF degradation efficiency of *Ralstonia* sp. SBUG290 was 0.0029 mmol/(L·h) [27], and *Pseudomonas putida* B2-6 degraded dibenzofuran (DBF) throug *Ralstoniah* co-metabolism at a rate of 0.0083 mmol/(L·h) [28]. The degradation efficiency increased rapidly during the first 48 h of incubation, reflecting bacterial adaptation to DBF as the sole carbon source (including induction of degradation enzymes) and exponential cell growth. After 48 h, the rate of increase slowed markedly (Figure 1b). This slowing is not due to a cessation of bacterial adaptation, but rather to the depletion of the DBF substrate, possible accumulation of intermediate metabolites, and the transition of the culture into a stationary phase. Therefore, 48 h was determined as the optimal incubation duration for subsequent experimental optimization.

The results demonstrated that strain FM-2 exhibited a broad temperature adaptability, with its DBF degradation efficiency remaining above 60% within the temperature range of 15–35 °C (Figure 1c). Following a 48-h cultivation period with a DBF concentration of 600 mg/L, the strain demonstrated a degradation efficiency of 67%, even at a low temperature of 15 °C. This finding possesses great research significance. Few DBF-degrading strains can stably maintain degradation efficiency at low temperatures, and strain tolerance performance also varies greatly under different pollutant concentrations. Representative cold-tolerant DBF-degrading bacteria include *Pseudomonas* sp. C3211 [29] and *Sphingobium* sp. BS19 [30]. Low temperature usually inhibits microbial metabolic activity, while excessively high DBF concentration generates toxic stress to bacterial cells; the synergistic restriction of the two factors jointly affects the actual biodegradation capacity of strains. This finding differs from the temperature preference of most reported PAH-degrading bacteria. The majority of these bacteria exhibit optimal degradation at moderate to high temperatures (typically 25–37 °C) and lose most of their activity below 20 °C. In contrast, strain FM-2 retained 67% of its degradation efficiency at 15 °C (Figure 1c), indicating a rare low-temperature adaptation capacity. The results of this study align with those of previous research reported by Saranya Kuppusamy et al. [31], who confirmed that *Sphingomonas* strains possess conserved DBF degradation pathways, prominent stress resistance and typical co-metabolic degradation characteristics at low temperatures. Furthermore, an increase in temperature to 20–35 °C resulted in a corresponding rise in the degradation efficiency to over 75%. This finding aligns with the conclusions of the study by Boopathy et al. [32], which demonstrated that by increasing the temperature appropriately, there was potential to enhance the PAH degradation capacity of degrading bacteria. The strain FM-2 exhibited the most pronounced degradation efficiency at 25 °C, suggesting that this temperature is optimal for its metabolic activity. Consequently, 25 °C was identified as the optimal cultivation temperature for subsequent experiments.

As shown in Figure 1d, strain FM-2 maintained a DBF degradation efficiency above 75% within the pH range of 5–9, indicating its excellent pH tolerance. The maximum degradation efficiency of 91.8% was obtained at pH 6.0, suggesting great application potential for this strain. The degradation efficiency exhibited a precipitous decline when the pH exceeded 9.0, concomitant with a near-complete cessation in bacterial growth. This phenomenon may be ascribed to the inactivation of PAH-degrading enzymes in strongly alkaline conditions. Conversely, when the pH was below 7.0, the degradation efficiency persisted at a level exceeding 80%. In comparison, *Pseudomonas* sp. ISTDF1 [33] and *Terrabacter* sp. DBF63 [34] exhibited inhibited or even undetectable DBF degradation at pH 6.0, along with retarded growth and cell death. Strain FM-2 possessed superior degradation performance and was more adaptable to DBF biodegradation in acidic environments. These characteristics further broaden its application prospects in low-temperature and high-acid contaminated sites.

As the salt concentration increased, a gradual decline in the DBF degradation efficiency was observed (Figure 1e). When the NaCl concentration was within 1%, the degradation efficiency remained above 65%, while a significant decline occurred once the concentration exceeded 1%. Accordingly, strain FM-2 is applicable to the bioremediation of contaminated environments with low salinity.

#### 3.1.2. Intermediate Product Identification and Degradation Pathway Analysis

The microbial degradation of dibenzofuran (DBF) is predominantly aerobic. The key initial step involves a dioxygenation reaction, in which molecular oxygen is introduced into the lateral or angular carbon atoms of the benzene ring to realize the oxidation and mineralization of DBF [35]. According to different oxygenation sites, the metabolic pathways can be divided into angular oxidation and lateral oxidation [17,36]. Among the currently isolated DBF-degrading microorganisms, angular dioxygenation is widely distributed, confirming that this pathway serves as the dominant route for microbial DBF degradation.

**Figure 2:**
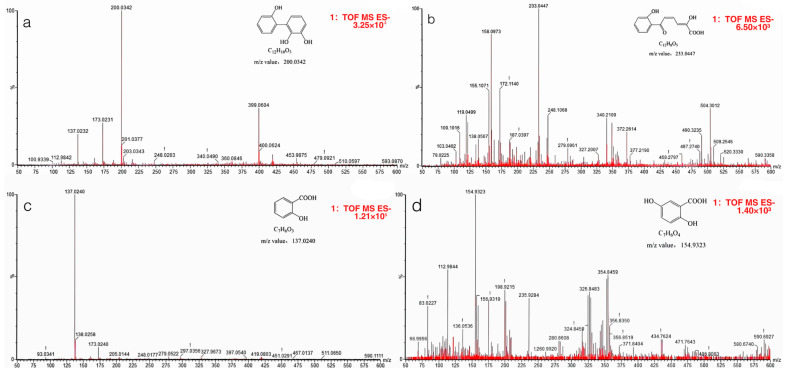
Results of the UPLC-MS/MS analysis of DBF metabolites produced in FM-2. (**a**) 2,2′,3-Trihydroxybiphenyl; (**b**) 2-Hydroxy-6-(2-hydrophenyl)-6-oxo-2,4-hexadienoic acid; (**c**) Salicylic acid; (**d**) Gentisic acid.

**Figure 3:**
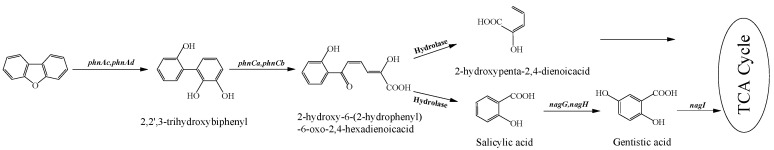
DBF degradation pathway of strain FM-2.

HPLC-MS was applied to elucidate the degradation pathway of DBF by strain FM-2. Based on comparative analysis of the extracted metabolites, four degradation intermediates were identified, namely 2,2′,3-trihydroxybiphenyl, 2-hydroxy-6-(2-hydrophenyl)-6-oxo-2,4-hexadienoic acid, salicylic acid, and gentisic acid (Figure 2). Previous genomic analysis in our laboratory confirmed that strain FM-2 harbors seven oxygenase-coding genes: phnAc and phnAd (encoding PAH dioxygenase), nagG and nagH (encoding salicylate 5-hydroxylase), phnCa and phnCb (encoding aromatic ring-opening dioxygenase), as well as nagI (encoding gentisate 1,2-dioxygenase) [26]. Thus, the metabolic pathway of DBF degradation by strain FM-2 was proposed. DBF is hydroxylated under the catalysis of dioxygenase encoded by phnAc and phnAd, followed by ring cleavage to produce 2,2′,3-trihydroxybiphenyl. Subsequently, this intermediate is oxidized by the ring-opening dioxygenase phnCa and phnCb to form 2-hydroxy-6-(2-hydrophenyl)-6-oxo-2,4-hexadienoic acid. After further hydrolysis, 2-hydroxypenta-2,4-dienoic acid and salicylic acid are generated. 2-Hydroxypenta-2,4-dienoic acid can directly enter the tricarboxylic acid cycle. In bacterial cells, salicylic acid is converted into catechol or gentisic acid via the hydroxylase encoded by nagG and nagH. Gentisic acid is further degraded by gentisate 1,2-dioxygenase (encoded by nagI) and ultimately enters the tricarboxylic acid cycle for subsequent metabolic processes (Figure 3).

### 3.2. Effect of Heavy Metal Cd(II) in LMM on DBF Degradation

After 7 days of incubation in LMM medium with 600 mg/L DBF as the sole carbon source and supplemented with different concentrations of Cd(II), the results showed that the MIC value of strain FM-2 against Cd(II) was 2000 mg/L (Table S2). This value was markedly higher than the reported MIC of 314 mg/L for *Pseudomonas* strain K1 described by Wang et al. [37] and 674 mg/L for *Flavobacterium* sp. PMSZPI documented by Macmillan et al. [38]. ByIn comparison, typical cadmium(II) concentrations in contaminated soil and industrial effluent range from a few milligrams per liter to approximately 100–200 mg/L. Consequently, the MIC of strain FM-2 is several orders of magnitude higher than common environmental levels. This indicates that its cadmium(II) tolerance far exceeds the concentrations found at most co-contaminated sites.

**Figure 4:**
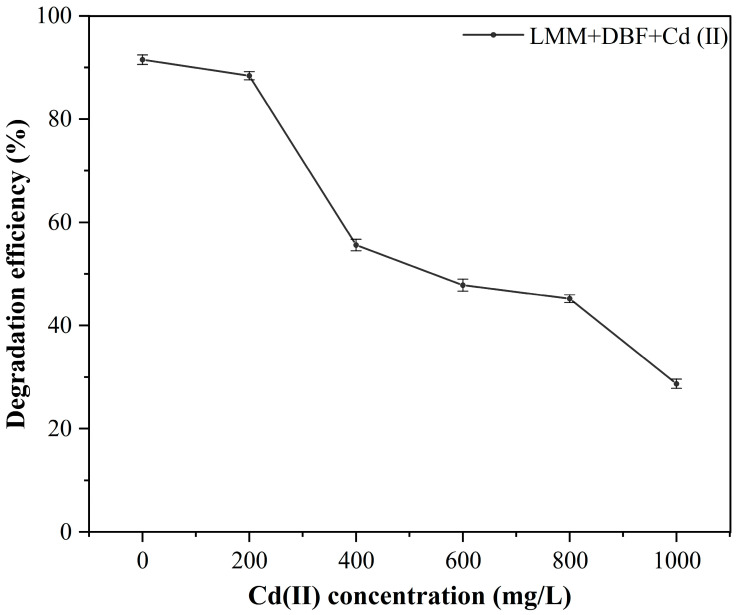
Effect of Cd(II) on DBF degradation within 7d.

As demonstrated in Figure 4, the DBF degradation efficiency remained above 85% when the Cd(II) concentration was no greater than 200 mg/L. This finding suggests that low concentrations of Cd(II) do not exert a clear toxic effect on the DBF-degrading enzyme system of strain FM-2. The strain was observed to typically utilize DBF as the carbon source for growth and metabolism, and its degradation activity was found to be largely unaffected. An increase in the concentration of Cd(II) from 200 mg/L to 400 mg/L resulted in a substantial decline in the degradation efficiency, which dropped to approximately 55%. It was hypothesized that under low cadmium stress (≤200 mg/L), the removal of Cd(II) by FM-2 primarily relies on intracellular bioaccumulation. Conversely, extracellular accumulation emerged as the predominant removal pathway at elevated Cd(II) concentrations (>200 mg/L), and the extracellular Cd(II) accumulation capacity exhibited an increase in proportion to the rise in initial Cd(II) levels [39]. With the further increase in Cd(II) concentration to 800 mg/L, the DBF degradation efficiency of strain FM-2 decreased to 45%, yet still remained at a relatively high level. These findings confirm the strong Cd(II) tolerance of strain FM-2. Consequently, FM-2 has been identified as having considerable potential for application in the bioremediation of sites contaminated with a combination of heavy metals and polycyclic aromatic hydrocarbons.

#### 3.2.1. SEM-EDS Analysis

**Figure 5:**
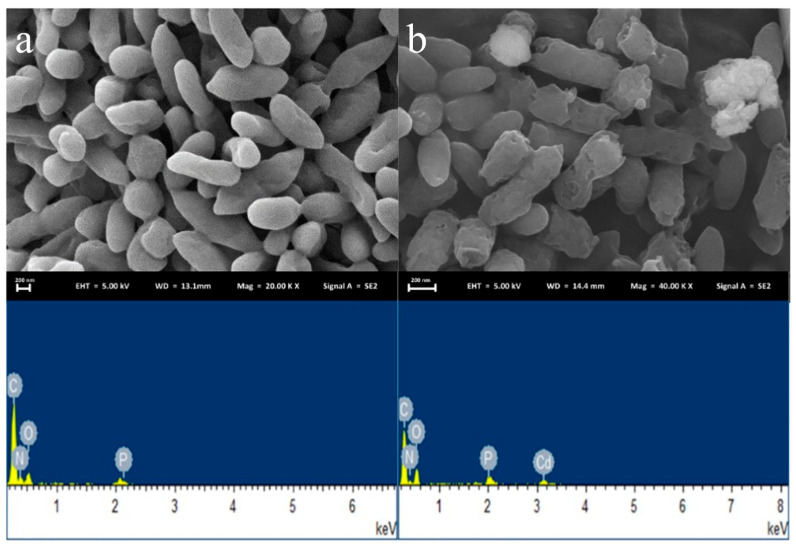
SEM-EDS of strain FM-2. (**a**) Control and (**b**) after Cd(II) adsorption.

Scanning electron microscopy coupled with energy dispersive X-ray spectroscopy (SEM-EDS) was utilized to elucidate the morphological disparities of strain FM-2 cells between the control group and the Cd(II)-biosorbed group. Bacterial cells that had not undergone Cd(II) treatment exhibited a short rod-shaped structure with a smooth surface and loose cell aggregation (Figure 5a). Conversely, after exposure to Cd(II), the surface of strain FM-2 exhibited a marked change, becoming rough and uneven with the presence of clearly visible porous structures (Figure 5b). This finding is indicative of the destructive effect of Cd(II) on the cell surface microstructure. Energy dispersive spectroscopy (EDS) analysis further verified the distribution of Cd(II) on the surface and inside bacterial cells. The heavy metal tolerance mechanism of FM-2 may be attributed to the precipitation of organic functional groups or the chelation of metal ions caused by cadmium deposition on the cell surface [40]. Despite the ambiguity surrounding the precise regulatory mechanism, these findings substantiate the notion that exogenous Cd(II) influences the surface morphology of bacterial cells [41] and exerts toxic stress on microbial cells.

#### 3.2.2. FTIR Spectroscopy Analysis

**Figure 6:**
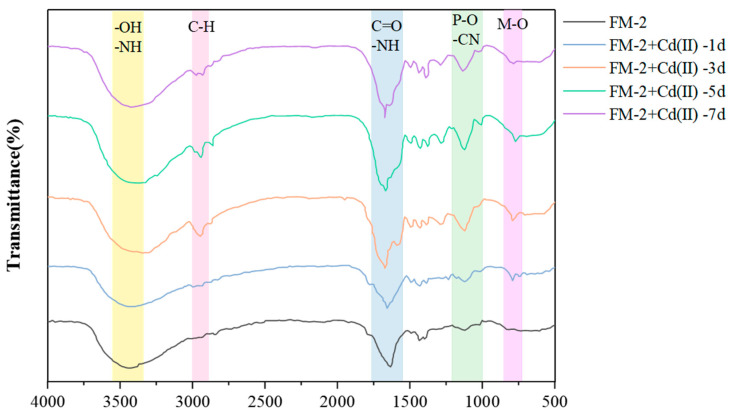
The effects of Cd(II) on FM-2 were analyzed by Fourier transform infrared spectroscopy (FTIR).

The surface functional groups of strain FM-2 after Cd(II) biosorption were analyzed within the wavenumber range of 500–4000 cm^−1^. As shown in Figure 6, a broad absorption band between 3600 and 3300 cm^−1^, assigned to hydroxyl groups, was detected in FM-2. With the extension of incubation time, this characteristic peak shifted toward 3300 cm^−1^, which might result from the stretching vibration of -OH bonds caused by the complexation between hydroxyl groups and metal ions [42,43]. Weak absorption peaks were observed near 2800–3000 cm^−1^, which were speculated to correspond to the C-H stretching vibration of methylene groups [44,45]. The peaks appearing at 1550–1800 cm^−1^ were attributed to the complexation of amide groups (including -NH and C=O stretching vibrations) with metal ions [46]. The absorption peak at 1160 cm^−1^ was ascribed to the C-N stretching vibration of amino groups, while the peak at 1070 cm^−1^ corresponded to the stretching vibration of P-O-C and partial P-O bonds in organophosphate groups [47]. A weak peak detected at 738 cm^−1^ appeared temporarily and then disappeared, suggesting that -CH groups could complex with metal ions to form stable δ(O-M-O) and δ(M-O) bonds, thereby inducing the displacement of stretching vibrations [48]. In conclusion, the findings of the FTIR spectral analysis indicated that the interaction between bacterial cells and Cd(II) ions was predominantly influenced by the presence of organophosphate, amide, and carboxyl groups.

#### 3.2.3. ICP-OES Analysis

**Figure 7:**
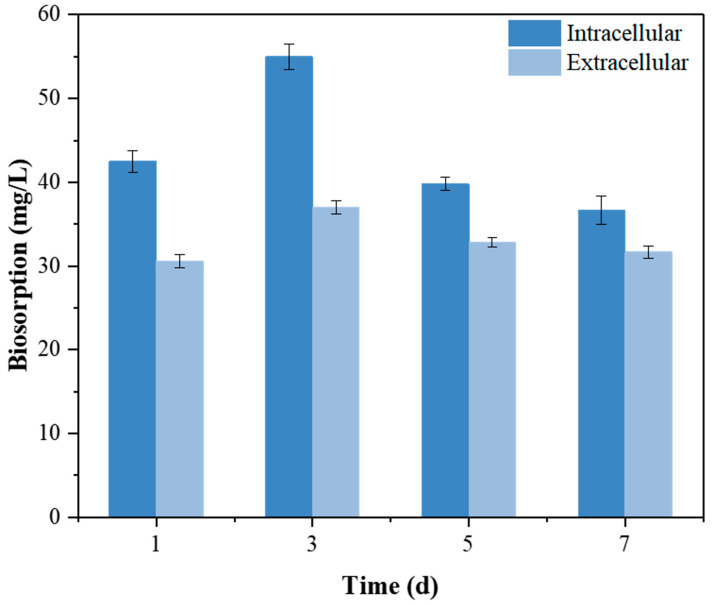
Adsorption efficiency of FM-2 for Cd(II).

Inductively coupled plasma-optical emission spectrometry (ICP-OES) enables the efficient and accurate determination of intracellular and extracellular heavy metal concentrations in microbial cells, which is essential for elucidating the complex mechanism of heavy metal bioaccumulation by strains. After strain FM-2 was cultured with 200 mg·L^−1^ Cd(II) for 1, 3, 5, and 7 days, the intracellular and extracellular Cd(II) bioaccumulation contents were determined. The results revealed that both intracellular and extracellular Cd(II) bioaccumulation reached the maximum values on the third day, with an intracellular content of 54.99 mg/L and extracellular content of 36.99 mg/L, followed by a gradual decline on the fifth and seventh days (Figure 7). Moreover, the intracellular Cd(II) accumulation was consistently higher than the extracellular accumulation throughout the seven-day incubation period. Combined with the detection of characteristic Cd peaks in the EDS spectra described in Section 3.2.1, this direct visual evidence confirms that Cd(II) is not only adsorbed onto the cell surface but is also internalized into the cytoplasm. The combination of EDS and ICP-OES provides strong evidence that the FM-2 strain is capable of the intracellular accumulation of Cd(II). As this strain is able to internalize Cd(II), it is speculated that the FM-2 strain may process Cd(II) via intracellular metabolic pathways [49]. After seven days of cultivation, the DBF degradation efficiency of FM-2 reached 89.7%. Compared with several reported cadmium-tolerant strains [50,51], FM-2 exhibited outstanding comprehensive performance under combined pollution stress.

### 3.3. Transcriptomic Analysis of DBF Degradation by Burkholderia *sp.* FM-2 Under Cd(II) Stress

#### 3.3.1. Analysis of Differentially Expressed Genes in Strain FM-2

**Figure 8:**
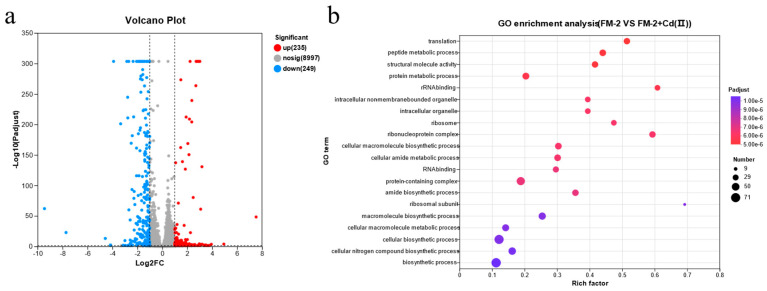
(**a**) Volcano plot. (**b**) GO enrichment analysis.

We analyzed the gene expression of strain FM-2 during the degradation of DBF after the addition of Cd(II) using transcriptome sequencing technology and conducted a comparative analysis of gene expression between the control group (without Cd(II)) and the experimental group (with 1000 mg/L Cd(II)), identifying differentially expressed genes using the screening criteria |log_2_FC| > 1 (i.e., FC > 2 or FC < 0.5) and *p*-value < 0.05. As shown in Figure 8a, a total of 484 genes exhibited significant expression changes following exposure to 100 mg/L Cd(II), including 235 genes that were found to be overexpressed and 249 genes that were found to be under expressed. The results indicate that Cd(II) stress triggered the stress response of strain FM-2 and induced corresponding metabolic regulation.

#### 3.3.2. GO Functional Annotation Analysis of Differentially Expressed Genes

To elucidate the mechanism of Cd(II) action during DBF degradation by strain FM-2, we focused on the GO functional annotations of both upregulated and downregulated genes, with relevant results presented in Figure 8b. A comparative analysis between the Cd(II)-treated experimental group and the untreated control group revealed that genes with functional differences were highly concentrated in ribosomal components, translational processes, and RNA binding functions. This finding is consistent with previous reports in heavy metal toxicology research [52]. For instance, in *Pseudomonas putida* [53], Cd stress was shown to induce differential expression of ribosome-related genes, specifically downregulating the genes encoding ribosomal subunits. This phenomenon is believed to be a microbial defense strategy against heavy metal toxicity: by suppressing the synthesis of non-essential proteins, microorganisms can reduce the production of misfolded proteins and thereby alleviate intracellular oxidative stress. Furthermore, our study identified a significant enrichment of genes related to rRNA binding and rRNA processing, in addition to those encoding ribosomal proteins. This observation suggests that Cd stress not only disrupts the structural composition of ribosomes, but also directly interferes with rRNA maturation and ribosome assembly. Such interference may represent a more direct mode of toxic action.

#### 3.3.3. Effects of Cd(II) on the Expression of Genes Involved in the DBF Degradation Pathway

According to Table S3, the overall aerobic degradation pathway of DBF was fully activated in strain FM-2, and all core functional genes were significantly upregulated. The aromatic ring-hydroxylating dioxygenase α-subunit (OI25_RS20545), β-subunit (OI25_RS20550), and protocatechuate 3,4-dioxygenase (OI25_RS20535) are key enzymes responsible for initial oxygenation and ring cleavage, which mediate the preliminary hydroxylation of DBF and the scission of aromatic rings [54]. The expression levels of the genes encoding these enzymes increased markedly after Cd(II) exposure, with upregulation folds of 2.23, 2.37, and 2.85, respectively. In addition, the genes encoding aldehyde dehydrogenase (OI25_RS20520) and HcaB dehydrogenase (OI25_RS20555) were upregulated by 3.02-fold and 2.14-fold under Cd(II) stress. These enzymes catalyze the isomerization, dehydrogenation, and hydrolysis of intermediate metabolites, and facilitate the conversion of degradation products into central carbon metabolism [55]. In summary, the functional genes associated with DBF degradation maintained normal expression in strain FM-2. Through transcriptional regulation, the strain could initiate critical metabolic processes including aromatic ring cleavage and intermediate transformation, thereby sustaining efficient DBF degradation under cadmium stress.

#### 3.3.4. Effects of Cd(II) on the Expression of Heavy Metal Transport-Related Genes in Strain FM-2

As demonstrated in extant studies, microorganisms have the capacity to protect cells from heavy metal toxicity by regulating the transport mechanisms of heavy metal ions [56]. Three major heavy metal transport systems were identified in strain FM-2, namely the RND family, P-type ATPase family, and ABC transporters. The present results indicate that a series of genes encoding these three transport systems were significantly upregulated in strain FM-2, so as to maintain intracellular homeostasis (Table S4).

The RND-type efflux pump represents a crucial mechanism for Gram-negative bacteria to cope with heavy metal stress [57]. It has been established that the formation of a continuous transmembrane channel is enabled by the presence of periplasmic adaptor proteins, transmembrane permeases, and outer membrane channel proteins. This process facilitates the active efflux of heavy metal ions. In this study, the genes encoding various subunits of the RND efflux pump in strain FMBB2 were found to be expressed at varying levels. Multiple genes encoding periplasmic adaptor proteins (e.g., OI25_RS01715, OI25_RS01725, OI25_RS06725) were found to be upregulated by 2.45-fold, 2.37-fold, and 1.54-fold, respectively. This indicates that strain FM-2 alleviates heavy metal toxicity by activating the RND system. In addition, the gene OI25_RS01720, which encodes a CusA/CzcA family heavy metal efflux pump, was also significantly upregulated. Proteins of this family have been proven to mediate the efflux of multiple heavy metal ions such as Cd^2+^ and Cu^+^ [58], further confirming the broad-spectrum regulatory role of the RND system in the heavy metal tolerance of strain FM-2.

P-type ATP transporters are a category of unidirectional transport pumps that rely on ATP for energy to directly expel intracellular heavy metal ions out of cells [53]. In this study, all genes encoding heavy metal-transporting P-type ATPases in strain FM-2, including OI25_RS01690, OI25_RS12330, and OI25_RS31835, exhibited upregulated expression with fold changes ranging from 1.06 to 2.81. These genes are presumed to act as the core P-type ATPases responsible for Cd(II) efflux in this strain. The results demonstrate that heavy metal-resistant bacteria generally rely on multiple efflux systems to maintain intracellular metal ion homeostasis, which is consistent with the classical theory proposed by Nies [59]. P-type ATPases have been demonstrated to exert a direct efflux function in bacterial resistance to heavy metals such as Cd(II), and perform functional complementation with RND efflux pumps to rapidly reduce the concentration of free intracellular Cd(II).

ABC transporters have been shown to mediate the transmembrane transport of substrates driven by ATP hydrolysis. The metal ABC transport system is responsible for the recognition and capture of intracellular heavy metal ions via periplasmic substrate-binding proteins. These ions are subsequently transported through transmembrane permeases [60,61]. In strain FM-2, novel0877 and OI25_RS16140, which encode metal ABC transporter solute-binding proteins, were found to be overexpressed at 2.62-fold and 2.01-fold, respectively. Meanwhile, OI25_RS16130, responsible for encoding metal ABC transporter permease, also showed an upregulation of 1.42-fold. It has been hypothesized that strain FM-2 enhances the recognition and transport capacity of intracellular Cd(II) by activating the metal ABC transport system. Similar findings have been reported in the Cd stress response of *Pseudomonas putida* [53]. ABC transporters have been shown to participate in heavy metal tolerance through auxiliary efflux or intracellular compartmentalization, and function synergistically with the RND and P-type ATPase systems [62].

### 3.4. Verification of Differentially Expressed Genes (DEGs) in Strain FM-2 Under Cd(II) Exposure Using Quantitative Real-Time PCR (qRT-PCR)

**Figure 9:**
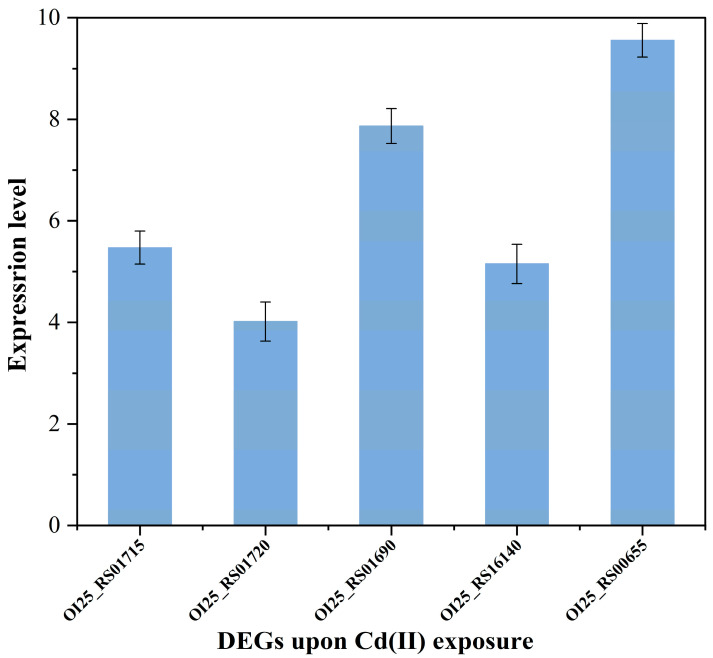
Expression levels of genes related to the efflux transporter and metal binding protein.

To validate the transcriptomic results, we used quantitative real-time PCR (qRT-PCR) to verify the expression levels of differentially expressed genes under Cd(II) stress. A total of five Cd(II) efflux transporter genes were identified, covering the RND efflux system, the P-type ATPase system, the ABC transport system, and copper-binding detoxification-related genes. These are the RND efflux transporter gene OI25_RS01715, the CusA/CzcA family heavy metal efflux pump gene OI25_RS01720, the heavy metal-transporting P-type ATPase gene OI25_RS01690, the ABC transporter gene OI25_RS16140, and the gene encoding the copper-binding protein CopC, OI25_RS00655. qRT-PCR results showed that the expression of all five selected target genes was significantly upregulated under Cd(II) stress conditions (Figure 9), and the fold changes in gene expression were fully consistent with the patterns observed in the transcriptomic sequencing data. The above validation results confirm the reliability of the transcriptome sequencing data. It was further verified that strain FM-2 can simultaneously activate multiple transport systems including RND efflux pumps, P-type ATPases, and ABC transporters. These systems work synergistically to efficiently expel intracellular Cd(II) and achieve detoxification, thereby endowing the strain with strong tolerance to heavy metals.

## 4. Conclusions

In this study, *Burkholderia* sp. FM-2 was confirmed to possess strong capacity for dibenzofuran biodegradation and high cadmium tolerance. Under optimized conditions of 600 mg/L initial DBF concentration, 25 °C, and pH 6.0, the strain achieved the maximum DBF degradation efficiency of 91.8% within 48 h, and exhibited excellent adaptability to low temperature and wide pH range. Low concentration of Cd(II) (≤200 mg/L) hardly inhibited its degradation performance, and the strain still maintained over 40% DBF degradation efficiency even under 800 mg/L Cd(II) stress, with the MIC value against Cd(II) reaching 2000 mg/L. Morphological and spectral characterization verified that Cd(II) stress changed the cell surface structure, and functional groups including amide, hydroxyl, and organophosphate groups participated in cadmium biosorption. ICP-OES results proved that intracellular accumulation was the dominant cadmium removal pattern of strain FM-2, and the maximum intracellular accumulation content reached 54.99 mg/L on the 3rd day of culture. Transcriptomic analysis revealed that 484 differentially expressed genes were induced under Cd(II) stress, including 235 upregulated and 249 downregulated genes. Core functional genes involved in the DBF aerobic degradation pathway were significantly upregulated with fold changes ranging from 2.14 to 3.02, ensuring stable metabolic degradation of DBF under heavy metal stress. Meanwhile, multiple heavy metal transport systems including RND efflux pumps, P-type ATPases, and ABC transporters were activated collaboratively to maintain intracellular ion homeostasis and enhance cadmium resistance.

This study systematically clarified the DBF degradation characteristics, metabolic pathways, and molecular response mechanisms of strain FM-2 under Cd(II) combined pollution, which provides reliable strain resources and a theoretical basis for the bioremediation of actual sites contaminated by PAHs and heavy metals.

## Data Availability

The original contributions presented in this study are included in the article/Supplementary Materials. Further inquiries can be directed to the corresponding authors.

## Supplementary Materials

The following supporting information can be downloaded at: https://www.mdpi.com/article/10.3390/microorganisms14061297/s1, Table S1: Primers employed to evaluate gene expression in response to cadmium exposure in strain FM-2; Table S2: Tolerance of strain FM-2 to heavy metal Cd(II); Table S3: Effects of Cd(II) on the Expression of Genes Involved in the DBF Degradation Pathway; Table S4: Effect of Cd stress on gene expression of Cd efflux transporter.

## References

[1] Sun Y., Wu S., Gong G. (2019). Trends of research on polycyclic aromatic hydrocarbons in food: A 20-year perspective from 1997 to 2017. Trends Food Sci. Technol..

[2] Paulik L.B., Smith B.W., Bergmann A.J., Sower G.J., Forsberg, N.D., Teeguarden J.G., Anderson K.A. (2016). Passive samplers accurately predict PAH levels in resident crayfish. Sci. Total Environ..

[3] Li R., Zhang J., Krebs P. (2021). Consumption- and Income-Based Sectoral Emissions of Polycyclic Aromatic Hydrocarbons in China from 2002 to 2017. Environ. Sci. Technol..

[4] Wild S.R., Jones K.C. (1995). Polynuclear aromatic hydrocarbons in the United Kingdom environment: A preliminary source inventory and budget. Environ. Pollut..

[5] Xia K., Ni Y., Zhan F., Song B., Ren Y., Sun Y., Gao Y., Cao R., Zhang Y., Chen J. (2020). Mechanistic aspects of polychlorinated dibenzo-p-dioxins and dibenzofurans (PCDD/Fs) formation from chlorine bleaching of non-wood pulp. J. Hazard. Mater..

[6] Bedient P.B., Rodgers A.C., Bouvette T.C., Tomson M.B., Wang T.H. (1984). Ground-water quality at a creosote waste site. Groundwater.

[7] Lee W.S., Chang-Chien G.P., Wang L.C., Lee W.J., Tsai P.J., Chen C.K. (2003). Emissions of polychlorinated dibenzo-p-dioxins and dibenzofurans from the incinerations of both medical and municipal solid wastes. Aerosol Air Qual. Res..

[8] Dash H.R., Das S. (2014). Bioremediation potential of mercury by *Bacillus* species isolated from marine environment and wastes of steel industry. Bioremediat. J..

[9] Biswas R., Sarkar A. (2019). Characterization of arsenite-oxidizing bacteria to decipher their role in arsenic bioremediation. Prep. Biochem. Biotechnol..

[10] Mohamed M.S.M., El-Arabi N.I., El-Hussein A., El-Maaty S.A., Abdelhadi A.A. (2020). Reduction of chromium-VI by chromium-resistant Escherichia coli FACU: A prospective bacterium for bioremediation. Folia Microbiol..

[11] Rehman K., Fatima F., Waheed I., Akash M.S.H. (2018). Prevalence of exposure of heavy metals and their impact on health consequences. J. Cell. Biochem..

[12] Jaishankar M., Tseten T., Anbalagan N., Mathew B.B., Beeregowda K.N. (2014). Toxicity, mechanism and health effects of some heavy metals. Interdiscip. Toxicol..

[13] Sun Q., Li Y., Shi L., Hussain R., Mehmood K., Tang Z., Zhang H. (2022). Heavy metals induced mitochondrial dysfunction in animals: Molecular mechanism of toxicity. Toxicology.

[14] Nnaji N.D., Anyanwu C.U., Miri T., Onyeaka H. (2024). Mechanisms of heavy metal tolerance in bacteria: A review. Sustainability.

[15] Rayaroth M.P., Marchel M., Boczkaj G. (2023). Advanced oxidation processes for the removal of mono and polycyclic aromatic hydrocarbons–A review. Sci. Total Environ..

[16] Bianchi D., Bosetti A., Cidaria D., Bernardi A., Gagliardi I., D’Amico P. (1997). Oxidation of polycyclic aromatic heterocycles by *Pseudomonas fluorescens* TTC1. Appl. Microbiol. Biotechnol..

[17] Wilkes H., Wittich R., Timmis K.N., Fortnagel P., Francke W. (1996). Degradation of chlorinated dibenzofurans and dibenzo-p-dioxins by *Sphingomonas* sp. strain RW1. Appl. Environ. Microbiol..

[18] Wang X., Che Y., Xu Y., Wu Y., Xu H., Li L. (2025). Mechanisms of nano zero-valent iron in enhancing dibenzofuran degradation by a *Rhodococcus* sp.: Trade-offs between ATP production and protection against reactive oxygen species. J. Hazard. Mater..

[19] Xu K., Wang H., Li P. (2021). The cadmium toxicity in gills of Mytilus coruscus was accentuated by benzo (a) pyrene of higher dose but not lower dose. Comp. Biochem. Physiol. Part C Toxicol. Pharmacol..

[20] Chiboub M., Jebara S.H., Abid G., Jebara M. (2020). Co-inoculation effects of *Rhizobium sullae* and *Pseudomonas* sp. on growth, antioxidant status, and expression pattern of genes associated with heavy metal tolerance and accumulation of cadmium in *Sulla coronaria*. J. Plant Growth Regul..

[21] Li J., Ou Y., Wang L., Zheng Y., Xu W., Peng J., Zhang X., Cao Z., Ye J. (2024). Responses of a PAH-Degrading Bacteria *Paraburkholderia Fungorum* JT-M8 to Cd(II) Under Oligotrophic Conditions. J. Hazard. Mater..

[22] Zhang J., Li Q., Zeng Y., Zhang J., Lu G., Dang Z., Guo C. (2019). Bioaccumulation and distribution of cadmium by *Burkholderia cepacia* GYP1 under oligotrophic condition and mechanism analysis at proteome level. Ecotoxicol. Environ. Saf..

[23] Ma J., Zhai Y., Cui Y., Gao G., Ying M., Zhao Y., Antunes A., Huang L., Li M. (2025). Study and Modification of the Polycyclic Aromatic Hydrocarbon Degradation Gene Cluster in *Burkholderia* sp. FM-2. Microorganisms.

[24] Zhai Y., Ma J., Gao G., Cui Y., Ying M., Zhao Y., Antunes A., Huang L., Li M. (2025). Enhancing Phenanthrene Degradation by *Burkholderia* sp. FM-2 with Rhamnolipid: Mechanistic Insights from Cell Surface Properties and Transcriptomic Analysis. Microorganisms.

[25] Liu X., Hu X., Cao Y., Pang W.J., Huang J.Y., Guo P., Huang L. (2019). Biodegradation of phenanthrene and heavy metal removal by acid-tolerant *Burkholderia fungorum* FM-2. Front. Microbiol..

[26] Ma J., Luo Z., Gao G., Cui Y., Ying M., Huang L., Li M. (2025). Biochar enhances the remediation of fluorene and Cd(II) co-contamination by *Burkholderia* sp. FM-2. Ecotoxicol. Environ. Saf..

[27] Becher D., Specht M., Hammer E., Francke W., Schauer F. (2000). Cometabolic degradation of dibenzofuran by biphenyl-cultivated *Ralstonia* sp. strain SBUG 290. Appl. Environ. Microbiol..

[28] Li Q., Wang X., Yin G., Gai Z., Tang H., Ma C., Deng Z., Xu P. (2009). New metabolites in dibenzofuran cometabolic degradation by a biphenyl-cultivated *Pseudomonas putida* strain B6-2. Environ. Sci. Technol..

[29] Jensen A.M., Finster K.W., Karlson U. (2003). Degradation of carbazole, dibenzothiophene, and dibenzofuran at low temperature by *Pseudomonas* sp. strain C3211. Environ. Toxicol. Chem..

[30] Sato K., Take S., Ahmad S.A., Gomez-Fuentes C., Zulkharnain A. (2023). Carbazole Degradation and Genetic Analyses of *Sphingobium* sp. Strain BS19 Isolated from Antarctic Soil. Sustainability.

[31] Kuppusamy S., Thavamani P., Megharaj M., Lee Y.B., Naidu R. (2016). Polyaromatic hydrocarbon (PAH) degradation potential of a new acid tolerant, diazotrophic P-solubilizing and heavy metal resistant bacterium *Cupriavidus* sp. MTS-7 isolated from long-term mixed contaminated soil. Chemosphere.

[32] Boopathy R. (2000). Factors limiting bioremediation technologies. Bioresour. Technol..

[33] Jaiswal P.K., Kohli S., Gopal M., Thakur I.S. (2011). Isolation and characterization of alkalotolerant *Pseudomonas* sp. strain ISTDF1 for degradation of dibenzofuran. J. Ind. Microbiol. Biotechnol..

[34] Monna L., Omori T., Kodama T. (1993). Microbial degradation of dibenzofuran, fluorene, and dibenzo-p-dioxin by *Staphylococcus auriculans* DBF63. Appl. Environ. Microbiol..

[35] Sakaki T., Munetsuna E. (2010). Enzyme systems for biodegradation of polychlorinated dibenzo-p-dioxins. Appl. Microbiol. Biotechnol..

[36] Jin S., Zhu T., Xu X., Xu Y. (2006). Biodegradation of Dibenzofuran by *Janibacter terrae* Strain XJ-1. Curr. Microbiol..

[37] Wang N., Wang Y., Li B., Huang F., Sun C., Li X., Zhao R., Wang Y. (2023). Characteristics of a copper-cadmium tolerant strain screened from tailings and its potential in remediation of heavy metal contaminated soil. Water Air Soil Pollut..

[38] Nongkhlaw M., Joshi S.R. (2019). Molecular insight into the expression of metal transporter genes in *Chryseobacterium* sp. PMSZPI isolated from uranium deposit. PLoS ONE.

[39] Huang F., Guo C.L., Lu G.N., Yi X.Y., Zhu L.D., Dang Z. (2014). Bioaccumulation characterization of cadmium by growing *Bacillus cereus* RC-1 and its mechanism. Chemosphere.

[40] Vishan I., Sivaprakasam S., Kalamdhad A. (2017). Biosorption of lead using *Bacillus badius* AK strain isolated from compost of green waste(water hyacinth). Environ. Technol..

[41] Chatterjee S., Ghosh I., Mukherjea K.K. (2011). Uptake and removal of toxic Cr(VI) by *Pseudomonas aeruginosa*: Physicochemical and biological evaluation. Curr. Sci..

[42] Allievi M.C., Sabbione F., Prado-Acosta M., Palomino M.M., Ruzal S.M., Sanchez C. (2011). Metal biosorption by surface layer protein from *Bacillus* species. J. Microbiol. Biotechnol..

[43] Pangnanelli F., Papini P.M., Toro L., Trifoni M., Veglio F. (2000). Biosorption of metal ions on *Arthrobacter* sp.:biomass characterization and biosorption modeling. Environ. Sci. Technol..

[44] Zhang J.-H., Min H. (2010). Characterization of a multimetal resistant *Burkholderia fungorum* isolated from an e-waste recycling area for its potential in Cd sequestration. World J. Microbiol. Biotechnol..

[45] Vishan I., Laha A., Kalamdhad A.S. (2017). Biosorption of Pb(II) by *Bacillus badius* AK strain originating from rotary drum compost of water hyacinth. Water Sci. Technol..

[46] Seki H., Suzuki A., Maruyama H. (2005). Biosorption of chromium(VI) and arsenic(V) onto methylated yeast biomass. J. Colloid. Interf. Sci..

[47] Jiang W., Saxena A., Song B., Ward B.B., Beveridge T.J., Myneni S.C. (2004). Elucidation of functional groups in Gram-positive and Gram-negative bacterial surfaces using infrared spectroscopy. Langmuir.

[48] Kazy S.K., Das S.K., Sar P. (2006). Lanthanum biosorption by a *Pseudomonas* sp.: Equilibrium studies and chemical characterization. J. Ind. Microbiol. Biotechnol..

[49] Chiboub M., Saadani O., Fatnassi I.C., Abdelkrim S., Abid G., Jebara M., Jebara S.H. (2016). Characterization of Efficient Plant-Growth-Promoting Bacteria Isolated from *Sulla coronaria* Resistant to Cadmium and to Other Heavy Metals. C. R. Biol..

[50] Liang Y., Chen J.Q., Mei J., Chang J.J., Wang Q.Y., Wan G.S., Yin B.Y. (2019). Characterization of Cu and Cd biosorption by *Pseudomonas* sp. strain DC-B3 isolated from metal mine soil. Int. J. Environ. Sci. Technol..

[51] Huang H., Jia Q., Jing W., Dahms H.U., Wang L. (2020). Screening strains for microbial biosorption technology of cadmium. Chemosphere.

[52] Khan Z., Elahi A., Bukhari D.A., Rehman A. (2022). Cadmium sources, toxicity, resistance and removal by microorganisms-A potential strategy for cadmium eradication. J. Saudi Chem. Soc..

[53] Cánovas D., Cases I., De Lorenzo V. (2003). Heavy metal tolerance and metal homeostasis in *Pseudomonas putida* as revealed by complete genome analysis. Environ. Microbiol..

[54] Kahl S., Hofer B. (2003). A Genetic System for the Rapid Isolation of Aromatic-Ring-Hydroxylating Dioxygenase Activities. Microbiology.

[55] Shortall K., Djeghader A., Magner E., Soulimane T. (2021). Insights into aldehyde dehydrogenase enzymes: A structural perspective. Front. Mol. Biosci..

[56] Xia X., Wu S., Zhou Z., Wang G. (2020). Microbial Cd(II) and Cr(VI) resistance mechanisms and application in bioremediation. J. Hazard. Mater..

[57] Hassen B., Abbassi M.S. (2025). Molecular mechanisms of heavy metal resistance and cross-/co-resistance to antibiotics in *Pseudomonas aeruginosa*. Lett. Appl. Microbiol..

[58] Chettri U., Nongkhlaw M., Joshi S.R. (2023). Molecular evidence for occurrence of heavy metal and antibiotic resistance genes among predominant metal tolerant *Pseudomonas* sp. and *Serratia* sp. prevalent in the Teesta River. Curr. Microbiol..

[59] Silver S., Phung L.T. (1996). Bacterial heavy metal resistance: New surprises. Annu. Rev. Microbiol..

[60] Nies D.H., Silver S. (1995). Ion efflux systems involved in bacterial metal resistances. J. Ind. Microbiol..

[61] Song W.Y., Park J., Eisenach C., Maeshima M., Lee Y., Martinoia E. (2014). ABC transporters and heavy metals. Plant ABC Transporters.

[62] Wen S., Yin F., Liu C., Dang Y., Sun D., Li P. (2023). Integrated analysis of transcriptomic and protein-protein interaction data reveals cadmium stress response in *Geobacter sulfurreducens*. Environ. Res..

