# Supplementary material for: Transcriptomic Analysis of Dibenzofuran Degradation by Burkholderia sp. FM-2 Under Cd(II) Stress
Source: Microorganisms. 2026 Jun 9;14(6):1297. doi: 10.3390/microorganisms14061297 (PMC13303270; doi:10.3390/microorganisms14061297)
Supplement: Supplementary file 1 [file microorganisms-14-01297-s001.zip › microorganisms-4314708-supplementary.pdf]

[Supplementary information]

Table S1. Primers employed to evaluate gene expression in response to cadmium exposure in strain FM-2

Name of primers	Sequence (5'-3')
OI25_RS01715- F:	GGTGCTGTTTCTGACCGC
OI25_RS01715- R:	AGGCGTTCGCTTTCTTTG
OI25_RS01720- F:	TGCTGATTCTGCCGTTTC
OI25_RS01720- R:	GCTGCTGATGGCGATGTT
OI25_RS01690- F:	ACCGTGGATGTGTTTGGC
OI25_RS01690- R:	ATCACTTTGCCGTTGGTG
OI25_RS16140- F:	AGGATGTGGGCGATAAAC
OI25_RS16140- R:	CCAGCAGCAGAAACAGAA
OI25_RS00655- F:	GGGCGAAACCTTTACCTA
OI25_RS00655- R:	ATCAGGCTGCCATACACG

Table S2. Tolerance of strain FM-2 to heavy metal Cd(II)

**Metal Ions**	**Metal Ion Concentration (mg/L)**											**MIC (mg/L)**
	0	50	100	150	200	300	400	500	1000	1500	2000	
**Cd(II)**	++++	++++	++++	++++	++++	++++	++++	+++	++	+	-	2000

Note：- : $OD_{600} < 0.4$; + : $0.4 < OD_{600} < 0.7$; ++: $0.7 < OD_{600} < 1$; +++ : $1 < OD_{600} < 1.2$; ++++ : $OD_{600} > 1.2$

Table S3. Effects of Cd(II) on the Expression of Genes Involved in the DBF Degradation Pathway

Gene ID	Gene name	Description	Log_2FC
OI25_RS20545	OI25_RS20545	Aromatic ring dioxygenase alpha subunit	2.234
OI25_RS20550	OI25_RS20550	Aromatic ring dioxygenase beta subunit	2.369
OI25_RS20530	OI25_RS20530	Hypothetical aromatic degradation protein	3.186

OI25_RS20535	pcaH	Protocatechuate 3，4-dioxygenase	2.852
OI25_RS20540	nahD	2-hydroxychromene-2-carboxylate isomerase	2.699
OI25_RS23730	catA	Catechol 1，2-dioxygenase	1.088
OI25_RS24660	OI25_RS24660	Fe-dependent oxygenase/dioxygenase	0.515
OI25_RS24510	OI25_RS24510	Cytochrome P450 monooxygenase	0.485
OI25_RS23690	OI25_RS23690	Muconate cycloisomerase	0.99
OI25_RS26410	catB	Muconolactone delta-isomerase	0.762
OI25_RS19215	OI25_RS19215	Rieske iron-sulfur oxygenase	1.257
OI25_RS31105	OI25_RS31105	Alpha-ketoglutarate-dependent dioxygenase	0.681
OI25_RS17450	OI25_RS17450	Hydroxybenzoate dioxygenase	1.124
OI25_RS22165	OI25_RS22165	Flavin-containing monooxygenase	0.593
OI25_RS29010	OI25_RS29010	Quinone-dependent oxidoreductase	0.826
OI25_RS11630	OI25_RS11630	Putative ring-cleaving enzyme	0.664
OI25_RS33260	OI25_RS33260	Ferredoxin oxygenase reductase	1.035
OI25_RS20525	OI25_RS20525	Dihydrodipicolinate synthase	2.947
OI25_RS20520	OI25_RS20520	Aldehyde dehydrogenase	3.018
OI25_RS24655	OI25_RS24655	Alpha/beta hydrolase	0.588
OI25_RS28995	OI25_RS28995	Aldehyde oxidoreductase	0.651
OI25_RS15930	hcaB	3-phenylpropionate dehydrogenase	2.143
OI25_RS25700	OI25_RS25700	FAD-binding oxidoreductase	0.427
OI25_RS24515	OI25_RS24515	NAD(P)H-dependent oxidoreductase	0.573
OI25_RS30610	OI25_RS30610	Malic enzyme	0.743
OI25_RS18120	OI25_RS18120	Formate dehydrogenase	0.625
OI25_RS21455	OI25_RS21455	Ester hydrolase	0.521
OI25_RS27640	OI25_RS27640	Glutaryl-CoA dehydrogenase	0.837

OI25_RS32035	OI25_RS32035	Peroxidase	0.716
OI25_RS26025	OI25_RS26025	Thiol oxidoreductase	0.498
OI25_RS22735	OI25_RS22735	Pyruvate dehydrogenase	0.672
OI25_RS28150	OI25_RS28150	Ureohydrolase	0.412
OI25_RS16240	OI25_RS16240	Lipolytic hydrolase	0.557
OI25_RS31520	OI25_RS31520	Hydroxylase accessory protein	0.614
OI25_RS29645	OI25_RS29645	Coenzyme A hydrolase	0.463
OI25_RS27130	OI25_RS27130	Ketoreductase	0.529
OI25_RS20560	OI25_RS20560	Outer membrane protein OmpW	1.758
OI25_RS28450	OI25_RS28450	TolC outer membrane efflux protein	2.053
OI25_RS00640	OI25_RS00640	TolC family efflux transporter	1.855
OI25_RS01715	OI25_RS01715	RND superfamily transporter	1.292
OI25_RS01780	OI25_RS01780	RND permease	0.608
OI25_RS36330	OI25_RS36330	MFS multidrug transporter	0.534
OI25_RS29200	OI25_RS29200	MFS substrate carrier	0.44
OI25_RS34125	OI25_RS34125	ABC-type organic transporter	0.697
OI25_RS19740	OI25_RS19740	ABC ATP-binding protein	0.518
OI25_RS32650	OI25_RS32650	Heavy metal transporter	0.735
OI25_RS21010	OI25_RS21010	Porin outer membrane channel	0.814
OI25_RS23355	OI25_RS23355	Aromatic compound permease	0.562
OI25_RS30105	OI25_RS30105	Sulfate permease	0.436
OI25_RS17725	OI25_RS17725	Organic anion transporter	0.548
OI25_RS33710	OI25_RS33710	Lipopolysaccharide transporter	0.425
OI25_RS25165	OI25_RS25165	Divalent metal transporter	0.504
OI25_RS18335	OI25_RS18335	Efflux membrane protein	0.637
OI25_RS27415	OI25_RS27415	Inner membrane transport protein	0.481

OI25_RS20510	OI25_RS20510	Transcriptional regulatory protein	0.766
OI25_RS36535	OI25_RS36535	Stress-dependent transcription regulator	1.48
OI25_RS27875	OI25_RS27875	LysR family transcriptional regulator	0.535
OI25_RS38360	OI25_RS38360	LysR metabolic regulator	0.43
OI25_RS38450	OI25_RS38450	LysR pathway regulator	0.536
OI25_RS21635	OI25_RS21635	LysR aromatic regulator	0.546
OI25_RS08760	OI25_RS08760	LysR family transcription factor	0.613
OI25_RS13220	OI25_RS13220	AraC family regulator	0.472
OI25_RS26245	OI25_RS26245	TetR family regulator	0.416
OI25_RS15370	OI25_RS15370	Two-component response regulator	0.583
OI25_RS16495	OI25_RS16495	MarR family repressor	0.457
OI25_RS29420	OI25_RS29420	Crp carbon metabolism regulator	0.512
OI25_RS00195	OI25_RS00195	Multicopper oxidase	1.917
OI25_RS00645	OI25_RS00645	Copper-containing oxidase	2.182
OI25_RS28445	OI25_RS28445	Blue copper oxidase	2.375
OI25_RS26510	cydA	Cytochrome bd oxidase	1.622
OI25_RS26515	cydB	Cytochrome bd subunit	1.041
OI25_RS21940	OI25_RS21940	Glutaredoxin	0.449
OI25_RS30450	OI25_RS30450	Rubredoxin	0.561
OI25_RS23140	OI25_RS23140	Thioredoxin	0.475

Table S4. Effect of Cd stress on gene expression of Cd efflux transporter

Gene ID	Description	Fold change
OI25_RS12330	heavy metal translocating P-type ATPase	2.11
OI25_RS31835	heavy metal translocating P-type ATPase	1.51
OI25_RS02035	heavy metal translocating P-type ATPase	1.44

OI25_RS01690	heavy metal translocating P-type ATPase	2.81
OI25_RS01945	heavy metal translocating P-type ATPase	1.28
OI25_RS00200	heavy metal translocating P-type ATPase	1.06
OI25_RS01715	efflux RND transporter periplasmic adaptor subunit	2.45
OI25_RS01780	efflux RND transporter permease subunit	1.52
OI25_RS06725	multidrug efflux RND transporter permease subunit	1.54
OI25_RS09110	efflux RND transporter permease subunit	1.42
OI25_RS01775	efflux RND transporter periplasmic adaptor subunit	1.61
OI25_RS21575	efflux RND transporter periplasmic adaptor subunit	1.03
OI25_RS23490	efflux RND transporter periplasmic adaptor subunit	1.13
OI25_RS06070	MdtB/MuxB family multidrug efflux RND transporter permease subunit	1.10
OI25_RS27630	efflux RND transporter permease subunit	1.19
OI25_RS23855	efflux RND transporter periplasmic adaptor subunit	1.47
OI25_RS29885	efflux RND transporter permease subunit	1.20
OI25_RS00745	efflux RND transporter periplasmic adaptor subunit	1.44
OI25_RS35565	efflux RND transporter periplasmic adaptor subunit	1.38
OI25_RS06680	efflux RND transporter periplasmic adaptor subunit	1.41
OI25_RS19140	multidrug efflux RND transporter permease subunit	1.25
OI25_RS00740	efflux RND transporter permease subunit	1.23
OI25_RS23495	efflux RND transporter permease subunit	1.24
OI25_RS31080	efflux RND transporter periplasmic adaptor subunit	1.44

OI25_RS28785	efflux RND transporter periplasmic adaptor subunit	1.46
OI25_RS40375	efflux RND transporter periplasmic adaptor subunit	1.44
OI25_RS29890	efflux RND transporter periplasmic adaptor subunit	1.47
OI25_RS19135	efflux RND transporter periplasmic adaptor subunit	1.54
OI25_RS35560	efflux RND transporter permease subunit	1.29
OI25_RS27625	efflux RND transporter periplasmic adaptor subunit	1.47
OI25_RS28790	efflux RND transporter permease subunit	1.30
OI25_RS09105	efflux RND transporter periplasmic adaptor subunit	1.44
OI25_RS23500	efflux RND transporter permease subunit	1.32
OI25_RS06075	efflux RND transporter permease subunit	1.29
OI25_RS06675	efflux RND transporter permease subunit	1.40
OI25_RS40380	efflux RND transporter permease subunit	1.37
OI25_RS15820	copper homeostasis periplasmic binding protein CopC	2.13
OI25_RS00655	copper homeostasis periplasmic binding protein CopC	5.60
OI25_RS00090	copper homeostasis periplasmic binding protein CopC	1.04
OI25_RS01720	CusA/CzcA family heavy metal efflux RND transporter	1.46
novel0877	metal ABC transporter solute-binding protein	2.62
OI25_RS16140	metal ABC transporter solute-binding protein	2.01
OI25_RS16130	metal ABC transporter permease	1.42
novel0625	aspartate-alanine antiporter	2.51
OI25_RS01725	efflux RND transporter periplasmic adaptor	2.37

	subunit	
novel0621	MFS transporter	3.88
novel0269	MFS transporter	2.14
novel1028	efflux transporter outer membrane subunit	2.71
OI25_RS02085	cation-translocating P-type ATPase	1.61
OI25_RS22710	divalent metal cation transporter	1.78
